# Supplementary material for: Factors Associated with Burnout among Resident Physicians Responding to the COVID-19 Pandemic: A 2-Month Longitudinal Observation Study
Source: Int J Environ Res Public Health. 2022 Aug 7;19(15):9714. doi: 10.3390/ijerph19159714 (PMC9367700; doi:10.3390/ijerph19159714)
Supplement: Supplementary file 1 [file ijerph-19-09714-s001.zip › ijerph-1851092-supplementary.pdf]

Survey Assessing Burnout Over Time

Demographic and professional questions – included only on initial survey

Q1 Sex:

○ Female
○ Male
○ Other, please specify_________________

Q2 Age: Choose the range.

○ <=35 years
○ 35-50 years of age
○ 50 – 59 years of age
○ 60 + years of age

Q3 Race/ethnicity: (according to Census 2020)

○ American Indian or Alaska Native
○ Asian Indian
○ Black or African American
○ Chamorro
○ Chinese
○ Filipino
○ Japanese
○ Korean
○ Native Hawaiian
○ Other Asian, please specify
○ Other Pacific Islander, please specify
○ Samoan
○ Some other race, please specify_________________
○ Vietnamese
○ White

Q4 Are you Hispanic, Latinx or of Spanish origin? (according to Census 2020)

○ Yes
○ No

Q5 Please select your professional role:

○ Attending Physician
○ Fellow
○ Nurse
○ Nurse Practitioner
○ Physician Assistant
○ Resident PGY1
○ Resident PGY2
○ Resident PGY3
○ Resident PGY 4
○ Resident PGY 5

Q6 How long have you been practicing (in years)?

___ ○ **currently in training**

○**1-5 years,**

○**6-10 years,**

○**11-19 years**

○ **20 + ____**

Q7 Select your department:

○ Medicine - pulmonary critical care
○ Medicine - General Medicine
○ Medicine - any internal medicine subspecialty
○ Emergency medicine
○ Surgery
O OBGYN
○ Podiatry
○ Dental or dental subspecialty
○ Pediatrics
○ Other____

Questions administered on every survey.

Q8 Have you worked in a clinical environment in the last 24 hours?

○ Yes ○ No

Q9 Are you currently working outside your usual scope of practice or usual role?

○ Yes ○ No

Please reflect on how you are feeling today. How much do you disagree or agree with the following?

		Strongly Disagree	Disagree	Neither Agree nor Disagree	Agree	Strongly Agree
**Q10**	**I have the energy to deal with the demands of work.**	○	○	○	○	○
**Q11**	**I feel focused and capable.**	○	○	○	○	○
**Q12**	**I can remain calm, despite the situation.**	○	○	○	○	○
**Q13**	**I can take care of my own health and am sleeping and eating properly.**	○	○	○	○	○
**Q14**	**The hospital is providing enough support and information to help me do my job.**	○	○	○	○	○
**Q15**	**The work I am doing is meaningful and will help us all address this crisis.**	○	○	○	○	○
**Q16**	**I am learning and growing as a professional.**	○	○	○	○	○

Q17 Overall, based on your definition of burnout, how would you rate your level of burnout?

○ I enjoy my work. I have no symptoms of burnout.
○ Occasionally I am under stress, and I don't always have as much energy as I once did, but I don't feel burned out.
○ I am definitely burning out and have one or more symptoms of burnout, such as physical and emotional exhaustion.
○ The symptoms of burnout that I'm experiencing won't go away. I think about frustration at work a lot.
○ I feel completely burned out and often wonder if I can go on. I am at the point where I may need some changes or may need to seek some sort of help.

Question administered periodically (administered on first survey and every 4th survey)

Q18 Think about what you need to improve your well-being and performance. Check all that apply – Indicate the level of priority for those items that you choose.

	Moderate Priority	High Priority
**Supplies and Personal Protective Equipment (PPE)**	○	○
**Mandated breaks**	○	○
**Break spaces – for rest and food**	○	○
**Support and affirmation**	○	○
**Timely information**	○	○
**Mentorship and technical guidance**	○	○
**Spiritual Support**	○	○
**Mental health resources**	○	○

Other, please specify: ____
